# Epsom Salts an Anesthetic

**Published:** 1908-01

**Authors:** 


### Epsom Salts an Anesthetic.

Announcement of the discovery of a new anesthetic—safer, cheaper and simpler than any hitherto known—is about to be made by the Rockefeller Institute for Medical Research. Plans were under way yesterday for the spreading of the important tidings to the medical and surgical world.

The new anesthetic is nothing less than plain, common Epsom salts, or, to give it its scientific name, sulphate of magnesia. It was discovered by Dr. Samuel J. Meltzer, one of the Rockefeller experimenters. Its greatest value is that it permits any sort of an operation without any danger to the heart of the patient.

Either local or general anesthesia, it is said, may be produced by the injection of a 20 per cent solution of the familiar drug into the nerve tract governing the sensations of the part to be operated upon.

The beneficial effects of the discovery—the number of lives that may be saved through its use—will more than repay the $5,000,000 with which John D. Rockefeller has endowed the institute.

It will prevent deaths from the powerful reactionary influence of ether and chloroform, the institute workers believe. And it will give a cance of life to those whose fragile hearts will not stand the stress of the administration of those drugs.

Dr. Meltzer's experiments have been going on quietly for a long time. But such success has crowned his efforts. The *American* learned yesterday that it will not be long before he makes public the result of his many tests.

The present discovery came about almost entirely by accident.

the director explained. Dr. Meltzer was experimenting about two years ago with various simple drugs to find out "what place they had in the economy of the body." He injected for that purpose a solution of sulphate of magnesia into a dog.

It was noticeable after a few minutes that the animal grew quiet and listless. Its respiration grew slower and fainter. Finally the breathing apparently ceased.

That was a new bit of knowledge—that magnesium sulphate affected the respiratory system. Dr. Meltzer pondered over it. Then he got a bellows, and, with a tube, produced artificial respiration in the dog's lungs. The animal revived, scampered away, and still lives.

Further tests proved that with a weaker solution the dog's respiration returned naturally, and that it was completely insensible to pain during the period of anesthesia. While the dog was senseless, Dr. Meltzer found that its temperature neither rose nor fell. Temperature rises with ether or chloroform. He found, too, what was more important, that its heart beat with absolute, unswerving regularity. Irregular pulsation follows ether and chloroform.

Suspended animation was caused in this case by suspended respiration—in plain words, a practical stoppage of the breath.

Tests followed with other animals, among them a monkey. This latter experiment was performed before many members of the Academy of Medicine. The beast, ill-tempered and vicious, was calmed first by a small injection. Then it was anesthetized.

This suggested that the injection might be valuable in the treatment of insane persons. So experiments were performed—for the first time upon human beings—at a local institution for the insane. Several persons were rendered quiet and tractable without being made unconscious or without apparently losing any of their faculties.—*New York American.*

